# Isoflavones inhibit poly(I:C)-induced serum, brain, and skin inflammatory mediators - relevance to chronic fatigue syndrome

**DOI:** 10.1186/s12974-014-0168-5

**Published:** 2014-10-31

**Authors:** Magdalini Vasiadi, Jennifer Newman, Theoharis C Theoharides

**Affiliations:** Department of Integrative Physiology and Pathobiology, Molecular Immunopharmacology and Drug Discovery Laboratory, Tufts University School of Medicine, 136 Harrison Avenue, Boston, MA 02111 USA; Graduate Program in Pharmacology and Experimental Therapeutics, Sackler School of Graduate Biomedical Sciences, Tufts University, Boston, MA USA; Department of Neuroscience, Tufts University, School of Medicine, Boston, MA USA; Department of Internal Medicine, Tufts University School of Medicine and Tufts Medical Center, Boston, MA USA

**Keywords:** Brain, Fatigue, Inflammation, Isoflavones, Mast cells, Polyinosinic:polycytidylic acid, Skin, Stress, Swim

## Abstract

**Background:**

Chronic Fatigue Syndrome (CFS) is a neuroimmunoendocrine disease affecting about 1% of the US population, mostly women. It is characterized by debilitating fatigue for six or more months in the absence of cancer or other systemic diseases. Many CFS patients also have fibromyalgia and skin hypersensitivity that worsen with stress. Corticotropin-releasing hormone (CRH) and neurotensin (NT), secreted under stress, activate mast cells (MC) necessary for allergic reactions to release inflammatory mediators that could contribute to CFS symptoms.

**Objective:**

To investigate the effect of isoflavones on the action of polyinosinic:polycytidylic acid (poly(I:C)), with or without swim stress, on mouse locomotor activity and inflammatory mediator expression, as well as on human MC activation.

**Methods:**

Female C57BL/6 mice were randomly divided into four groups: (a) control/no-swim, (b) control/swim, (c) polyinosinic:polycytidylic acid (poly(I:C))/no swim, and (d) polyinosinic:polycytidylic acid (poly(I:C))/swim. Mice were provided with chow low or high in isoflavones for 2 weeks prior to ip injection with 20 mg/kg poly(I:C) followed or not by swim stress for 15 minutes. Locomotor activity was monitored overnight and animals were sacrificed the following day. Brain and skin gene expression, as well as serum levels, of inflammatory mediators were measured. Data were analyzed using the non-parametric Mann-Whitney *U*-test.

**Results:**

Poly(I:C)-treated mice had decreased locomotor activity over 24 hours, and increased serum levels of TNF-α, IL-6, KC (IL-8/CXCL8 murine homolog), CCL2,3,4,5, CXCL10, as well as brain and skin gene expression of *TNF*, *IL-6*, *KC (Cxcl1*, *IL8* murine homolog), *CCL2*, *CCL4*, *CCL5* and *CXCL10*. Histidine decarboxylase (*HDC*) and *NT* expression were also increased, but only in the skin, over the same period. High isoflavone diet reversed these effects.

**Conclusion:**

Poly(I:C) treatment decreased mouse locomotor activity and increased serum levels and brain and skin gene expression of inflammatory mediators. These effects were inhibited by isoflavones that may prove useful in CFS.

**Electronic supplementary material:**

The online version of this article (doi:10.1186/s12974-014-0168-5) contains supplementary material, which is available to authorized users.

## Background

Chronic Fatigue Syndrome (CFS) is a complex disease, which has also been called ‘neurasthenia’, ‘post viral fatigue’, and ‘chronic mononucleosis’ [1-3]. Its prevalence may be as high as 1% in the US population [4] with a female to male ratio of 4 to 1 [5]. CFS involves the muscular, nervous, hormonal and immune systems. Patients complain of overwhelming fatigue, sleep disturbances, malaise, muscle aches, gastrointestinal symptoms, dizziness on standing, and cognitive problems [6]. Clinical and subclinical viral infections have been suspected, but never confirmed [7,8].

CFS is often comorbid with other disorders, including fibromyalgia, Pelvic Bladder Syndrome/Interstitial Cystitis (PBS/IC), irritable bowel syndrome (IBS), and migraines [9], all of which are characterized by central nervous system (CNS) dysfunction [10], and worsened by stress [11-15]. Many CFS patients demonstrate abnormal hypothalamic-pituitary-adrenal (HPA) axis activity [16,17]. Anxiety is common in CFS [18] and patients are particularly vulnerable to stress [14]. Many CFS symptoms could derive from the possible release of inflammatory mediators that could affect brain function [19,20]. As of today, there are no FDA approved drugs for the treatment of CFS [21]; psychological, physical, and pharmacological interventions used currently are not very effective [22].

An abnormal immune component may be involved in CFS [23-25], but the neuroimmune and neuroendocrine interactions involved are still unknown [26]. Mast cells (MC) and their mediators have been implicated in all diseases that are comorbid with CFS [9]. There is higher number of skin MC in patients with CFS [27,28], and such patients also show increased skin hypersensitivity [29]. Furthermore, CFS also occurs more often in patients with chronic urticaria, that also involves MC [30]. In fact, there is hyperresponsiveness in the bronchi of CFS patients, implying MC activation [31].

Activated MC release a number of chemokines and cytokines that could contribute to CFS symptoms [32,33]. MC are located perivascularly in close proximity to neurons [34], especially in the diencephalon [35,36], where functional MC-neuron interactions have been documented [36,37] in response to corticotropin-releasing hormone (CRH) [38]. *In vivo* activation of MC by CRH is augmented by neurotensin (NT) [39]. Moreover, NT is induced in the hypothalamus in response to bacterial lipopolysaccharide (LPS) and regulates the HPA axis [40].

Unfortunately, there are neither effective CFS treatments nor human MC inhibitors clinically available that may also be used in CFS. Flavonoids are natural compounds with strong antioxidant and anti-inflammatory activity [41]. Certain flavonoids also inhibit MC [42] and have neuroprotective effects [41,43].

Here we report that treatment of mice with poly(I:C) results in reduced locomotor activity and increased serum levels, as well as brain and gene expression, of inflammatory mediators, all of which are reversed by treatment with the isoflavones daidzein and genistein.

## Methods

### Chemicals and reagents

Polyinosinic-polycytidylic acid-TLR3-based adjuvant, poly(I:C), HMW VacciGrade, (catalog# vac-pic) was purchased from Invivogen (San Diego, CA, USA). Substance P (SP, catalog# S6883), neurotensin (NT, catalog# N6383) and corticotropin-releasing hormone (CRF, catalog# C3042) were purchased from Sigma-Aldrich (St. Louis, MO, USA). Aliquots of the above were prepared according to the manufacturer’s instructions. Teklad lab animal diets (catalog# 2918X, and 2920X) were purchased from Harlan (Indianapolis, IN, USA).

### Animals

C57BL/6 female mice, nine to twelve weeks old, (Jackson Laboratories, Bar Harbor, ME, USA) were kept in virus-free sections of a modern animal facility and were allowed *ad libitum* access to food and water. They were maintained on a 14:10 hour light-dark cycle (the standard light-dark cycle used by the Department of Animal Health). Female mice were chosen because published reports indicate a female to male ratio of 4:1 [5], while the US Centers for Disease Control and Prevention (CDC) specify a female to male ratio of 4:1 [5]. Mice were kept in cages of five mice/cage until the day of the experiments. Both low and high isoflavone diets (2018X and 2020X) were sterile with similar ingredients other than isoflavone content. We monitored weight changes for 21 days prior to the beginning of the experiments. Only poly(I:C)/no swim-treated mice showed a slight decrease in weight change. Poly(I:C)/swim-treated mice, as well as their corresponding control mice, slightly increased their weight over the three-week observation period. However, by the end of this period, there was no statistical difference in weight change. The protocol was approved by Tufts Medical Center IACUC under number B 2011-88.

### Treatment conditions

Mice were provided with chow containing either non-detectable to low (ND-20 mg/kg, Teklad 2920X) or high (150 to 250 mg/kg, Teklad 2918X) isoflavone (daidzein plus genistein) levels for two weeks. Conditions included four groups: (a) control (normal saline intraperitoneal (ip) injection)/no swim, (b) control/swim, (c) poly(I:C)/no swim, and (d) poly(I:C)/swim (n = 5 to 7/group). Mice were injected ip with 20 mg/kg of poly(I:C) or normal saline the first day. Subsequently, they were subjected to swim for 15 minutes, individually in a transparent plastic cylindrical jar (17 cm × 25 cm) containing 15 cm-deep water at room temperature (23 ± 1°C). This approach reflects both exercise and the stress of water immersion. Mice were then placed individually into specific cages and locomotor activity was monitored overnight.

### Assessment of behavioral parameter-locomotor activity

After the experimental procedures, animals were placed individually into standard plastic housing cages with food and water available *ad libitum* and overnight locomotor activity (for a total of 16 hours) was monitored with the Neuroscience Behavior Core’s mouse SmartFrame® Cage Rack System (Kinder Scientific, Poway, CA, USA). This system consists of 20 PC-interfaced horizontal photobeam frames. The frame (containing 12 photocells; arranged on a 8 L × 4 W grid) surrounds one home cage environment and continuously tracks the animal’s movement. This fully automated system allows the user to quantify horizontal ambulation by counting breaks in infrared photocell beams using MotorMonitor® software (Hamilton-Kinder Scientific, Poway, CA, USA). Data were collected and subsequently analyzed in time bins (every hour) or as a total over the course of collection to the ‘Total Distance Travelled’ (in cm) parameter for each zone.

### Sample collection

Mice were euthanized 24 hours post poly(I:C) ip injection using isoflurane overdose and thoracotomy. Blood was collected by cardiac puncture and was used to determine inflammatory mediator levels in the serum. Brain (diencephalon) and skin (back shaved with a electric shaver the day before) samples were collected and immersed into RNAlater (catalog# AM7021) purchased from Invitrogen (Grand Island, NY, USA). Samples were stored at -80°C.

### Serum levels of inflammatory mediators

TNF-α, VEGFα, IL-1α, IL-1β, IL-4, IL-6, KC (IL-8/(CXC motif) ligand (CXC)L8 murine homolog, IL-9, IL-10, IL-12p70, IL-17, (CC motif) ligand (CCL)2, CCL3, CCL4, CCL5, CXCL10 and IFNγ mouse serum levels were determined using the MILLIPLEX MAP Mouse Cytokine/Chemokine Magnetic Bead Assay (MCYTOMAG-70 K custom made panel for the specific analytes mentioned above). Measurements were performed blindly by Millipore (St. Charles, MI, USA). Millipore's minimum assay reporting range for TNF-α and IL-6 levels was 32 pg/ml, for CCL2 was 800 pg/ml, while for CCL3 and CCL4 it was 160 pg/ml. Therefore, we consider all values < × pg/ml as equal to x pg/ml.

### Quantitative PCR

Total RNA from mouse tissues was extracted using RNeasy Plus Mini kit (catalog# 74134) and RNeasy Fibrous Tissue Mini Kit (catalog# 74704), purchased from QIAGEN (Valencia, CA, USA). Reverse transcription was performed with 300 ng of total RNA using the iScript cDNA synthesis kit (catalog# 170-8891) purchased from Bio-Rad (Hercules, CA, USA). Real-time quantitative polymerase chain reaction (qPCR) was carried out in a 7300 Sequence Detector, according to TaqMan Gene Expression Assay instructions from Life Technologies, Applied Biosystems (Grand Island, NY, USA) using Taqman primer/probe sets (Additional file 1: Table S1). Samples were analyzed for *Tnf (TNF-α), Il4, Il6 (IL-6)*, *KC (Cxcl1, IL-8* homolog in the mouse), *Ccl2 (CCL2)*, *Ccl4 (CCL4)*, *Ccl5 (CCL5)*, *Cxcl10 (CXCL10)*, histidine decarboxylase-*Hdc* (*HDC*), and *Nts (NT)* gene expression using *Gapdh* as internal control.Thermal cycling proceeded at 50°C for 2 minutes, 95°C for 10 minutes, 95°C for 15 seconds, for 40 cycles, and 60°C for 1 minute. Negative controls included samples with water instead of template. Assays were performed in triplicate for each data point. Results were normalized against the endogenous gene GAPDH and were expressed relative to the mean of the control for each gene (relative fold change).

### Statistical analysis

Data were analyzed using the non-parametric Mann-Whitney *U*-test. Results are presented as mean ± SE. Statistical significance is defined as *P* < 0.05. We analyzed separately the different treatment subgroups within each dietary group, using Mann-Whitney *U*-test to compare poly(I:C)/no swim and poly(I:C)/swim-treated mice with the control/no swim and control/swim-treated groups, accordingly. We then analyzed the same treatment subgroups between the two dietary groups, using Mann-Whitney *U*-test to compare, for example, poly(I:C)/swim-treated mice with low isoflavone diet to poly(I:C)/swim-treated mice that were provided with high isoflavone diet. Due to space limitations, we submitted figures composed of all the experimental conditions from the two different diets and all the statistical significant results were marked in these figures.

## Results

### Effect of poly(I:C) and isoflavones on locomotor activity

**Figure 1:**
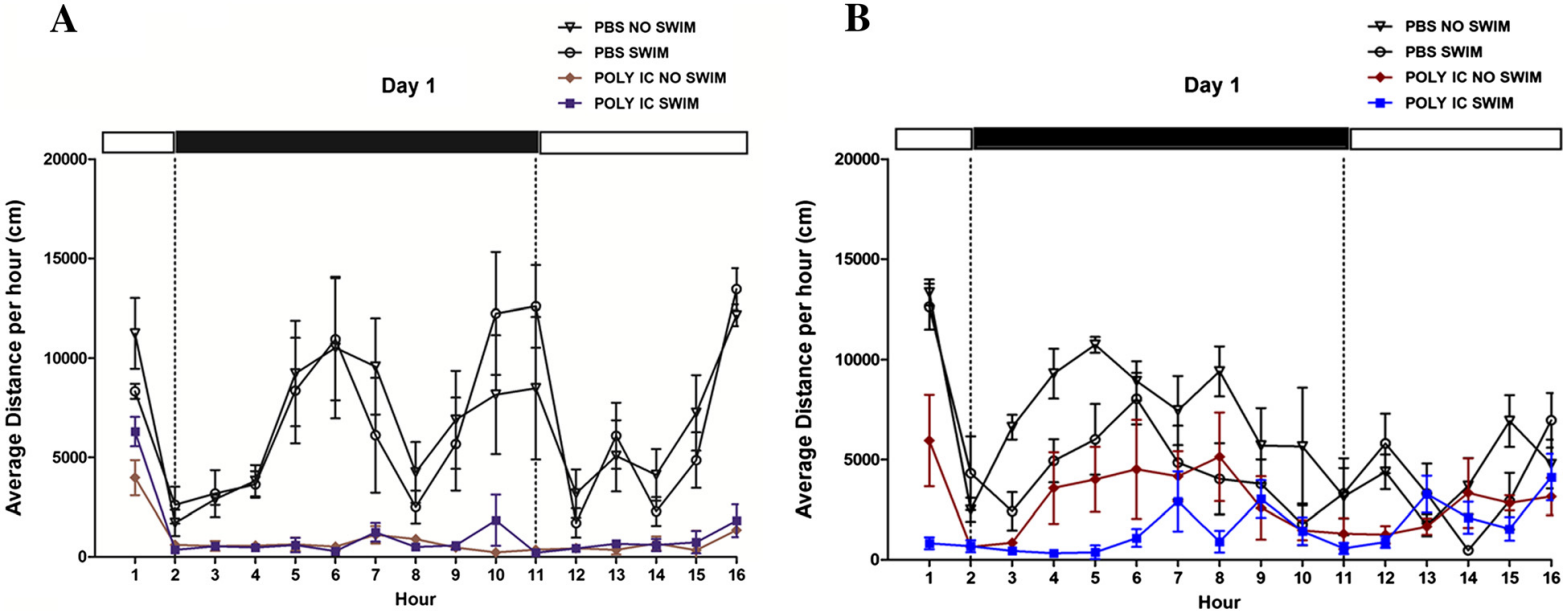
**Polyinosinic:polycytidylic acid (poly(I:C)) and isoflavone effect on mouse locomotor activity.** Mice were provided with either low (Figure 1 **A)** or high (Figure 1 **B)** isoflavone diet *ad libitum*. Mice were injected intraperitoneally (ip) with 20 mg/kg poly(I:C) and were forced to swim for 15 minutes. Following this, they were individually placed into specific cages and locomotor activity was monitored overnight.

Poly(I:C)/swim and poly(I:C)/no swim-treated mice on low isoflavones had reduced maximum (max) locomotor activity (over the 10-hour night period), denoted by the dark bar (*P* = 0.008 and *P* = 0.036) compared to the control/swim and control/no swim-treated mice, respectively (Figure 1A). High isoflavone chow reversed this decrease (Figure 1B), and the difference between the two diets was statistically significant (*P* = 0.032).

### Effect of poly(I:C) and isoflavones on serum inflammatory mediators

**Figure 2:**
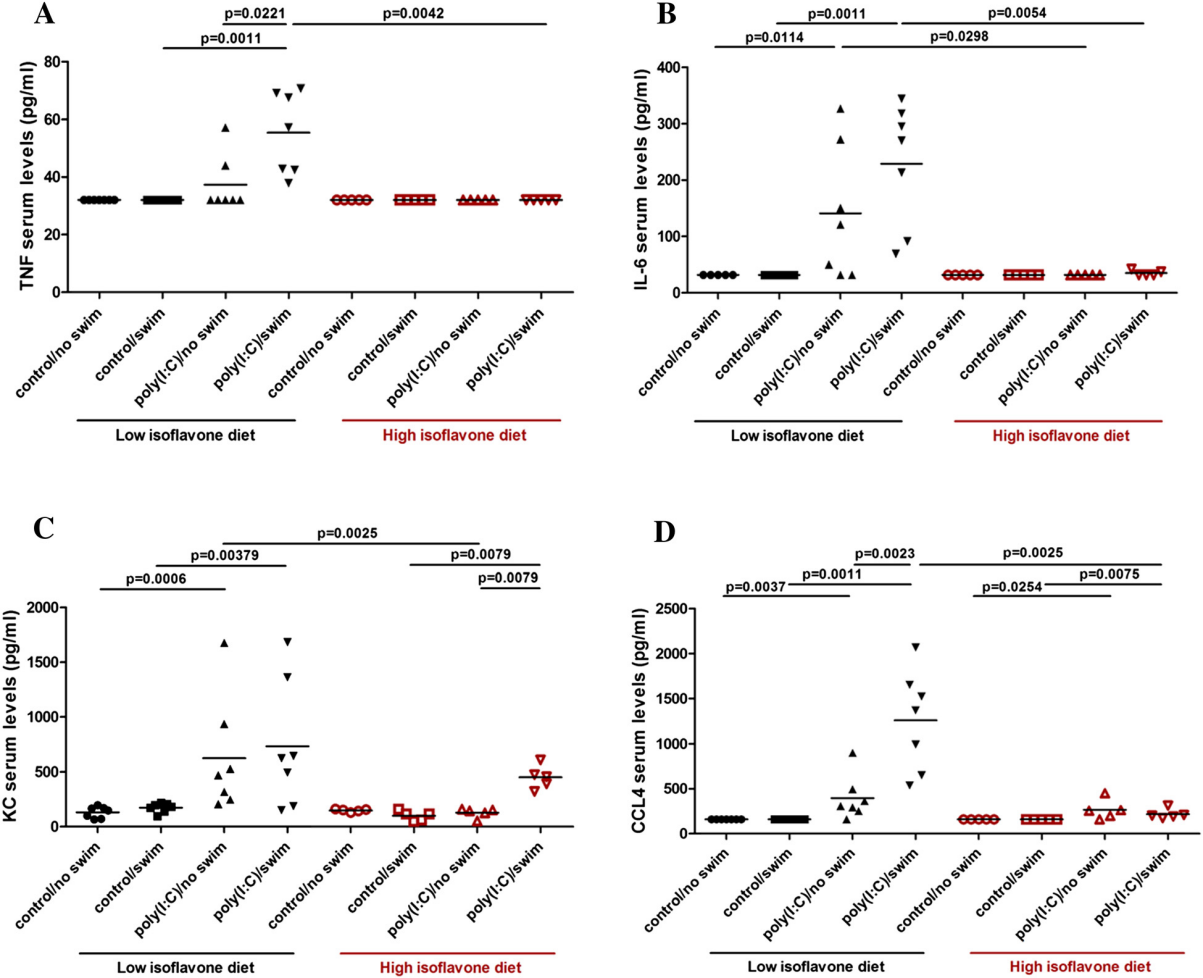
**Polyinosinic:polycytidylic acid (poly(I:C)) and isoflavone effect on serum levels of inflammatory mediators.** Mice were provided with either low or high isoflavone diet *ad libitum*. Mice were injected intraperitoneally (ip) with 20 mg/kg poly(I:C) and were forced to swim for 15 minutes. Following this, they were individually placed into specific cages and locomotor activity was monitored overnight. The next day, serum samples were collected and analyzed for TNF-α (Figure 2 **A)**, IL-6 (Figure 2 **B)**, KC (Figure 2 **C)**, and CCL4 (Figure 2 **D)** using the Milliplex microbead assay.

Poly(I:C)-treated mice on low isoflavones had increased serum levels of TNF-α (Figure 2A), IL-6 (Figure 2B), KC (Figure 2C), and CCL4 (Figure 2D), as well as CCL2, CCL3, CCL5, and CXCL10 (Additional file 2: Table S2). High isoflavones reduced the poly(I:C)-increased serum levels of all the inflammatory markers. All IFNγ, IL-1β, IL-4, IL-9, IL-10, IL-12p70, IL-17 and VEGFα serum levels were below the detection limit, while IL-1α serum levels were similar between the different treatment groups. Swim stress augmented the poly(I:C) effect on TNF-α, CCL4, and CCL5 serum levels.

More specifically, TNF-α serum levels in poly(I:C)/swim-treated mice on low isoflavones were increased (55 ± 14 pg/ml) compared to control/swim-treated mice (32 ± 0 pg/ml, *P* = 0.0011) and poly(I:C)/no swim-treated mice (37 ± 10 pg/ml,*P* = 0.0221). High isoflavones decreased these high TNF-α serum levels (32 ± 0 pg/ml, *P* = 0.0042) (Figure 2A). IL-6 serum levels in poly(I:C)-swim and poly(I:C)/no swim-treated mice on low isoflavones were also increased (229 ± 110 pg/ml and 141 ± 11 pg/ml) compared to the corresponding control/swim and control/no swim mice on low isoflavones (32 ± 0 pg/ml, *P* = 0.0011 and 32 ± 0 pg/ml, *P* = 0.0114) (Figure 2B). High isoflavones reduced IL-6 serum levels of poly(I:C)/swim-treated mice (35 ± 5 pg/ml, *P* = 0.0054) and poly(I:C)/no swim-treated (32 ± 0 pg/ml, *P* = 0.0298) compared to the corresponding groups on low isoflavones (Figure 2B).

Similarly, KC serum levels in poly(I:C)-treated mice on low isoflavones were increased (735 ± 579 pg/ml and 626 ± 523 pg/ml) compared to the corresponding controls (173 ± 43 pg/ml, *P* = 0.0379 and 132 ± 52 pg/ml, *P* = 0.0006, respectively) (Figure 2C). Although KC serum levels in poly(I:C)/swim-treated mice on high isoflavones were also increased (451 ± 107 pg/ml) compared to control poly(I:C)/swim-treated mice (100 ± 45 pg/ml, *P* = 0.0079), high isoflavone diet decreased KC serum levels in poly(I:C)/no swim-treated mice (128 ± 43 pg/ml, *P* = 0.0025) compared to those on low isoflavones (Figure 2C).

Moreover, CCL4 serum levels were increased in poly(I:C)-treated mice on low isoflavones (1,256 ± 556 pg/ml and 395 ± 245 pg/ml) compared to the corresponding controls (160 ± 0 pg/ml, *P* = 0.0011 and 160 ± 0 pg/ml, *P* = 0.0037, respectively) (Figure 2D). Swim further increased CCL4 serum levels (1,256 ± 556 pg/ml, *P* = 0.0023). Although CCL4 serum levels in poly(I:C)-treated mice on high isoflavones were also increased (217 ± 55 pg/ml and 265 ± 111 pg/ml) compared to their controls (160 ± 0 pg/ml, *P* = 0.0075 and 160 ± 0 pg/ml, *P* = 0.0254, respectively)., high isoflavones decreased CCL4 serum levels in poly(I:C)/swim-treated mice (217 ± 55 pg/ml, *P* = 0.0025) (Figure 2D).

Poly(I:C) treatment also increased CCL2, CCL3, CCL5 and CXCL10 serum levels and high isoflavones reduced those increases (Additional file 3).

### Effect of poly(I:C) and isoflavones on brain gene expression of inflammatory mediators

**Figure 3:**
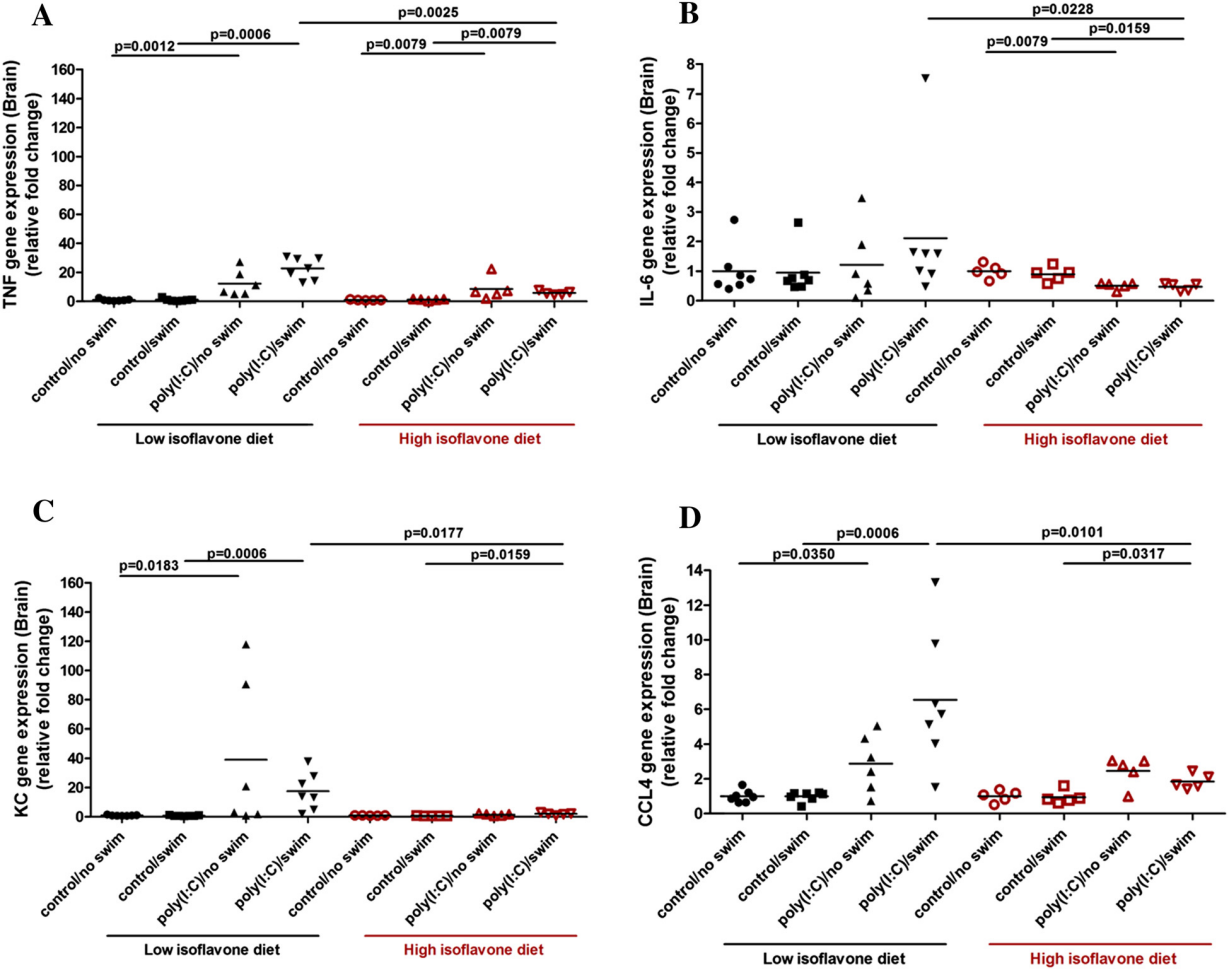
**Polyinosinic:polycytidylic acid (poly(I:C)) and isoflavone effect on brain gene expression of inflammatory mediators.** Mice were provided with either low or high isoflavone diet *ad libitum*. Mice were injected intraperitoneally (ip) with 20 mg/kg poly(I:C) and were forced to swim for 15 minutes. Following this, they were individually placed into specific cages and locomotor activity was monitored overnight. The next day, brain samples were collected and analyzed for *TNF-α* (Figure 3 **A)**, *IL-6* (Figure 3 **B)**, *KC* (Figure 3 **C)**, and *CCL4* (Figure 3 **D)** brain gene expression using qPCR.

*TNF-α* (Figure 3A), *IL-6* (Figure 3B), *KC* (Figure 3C), *CCL4* (Figure 3D), as well as *CCL2*, *CCL5* and *CXCL10* brain gene expression were increased in the poly(I:C)-treated mice. High isoflavones reduced the increased *TNF*, *IL6*, *KC*, *CCL4* and *CCL2* brain gene expression. There was no difference in *HDC* and *NT* gene expression between the different treatment groups (Additional file 4: Table S3).

More specifically *TNF-α* gene expression was increased in the brain of poly(I:C)-treated mice on low isoflavones (relative fold change of 22 ± 7 and 12 ± 9) compared to their controls (1.1 ± 0.9, *P* = 0.0006 and 1 ± 0.7, *P* = 0.0012, respectively) (Figure 3A). Although poly(I:C)-treated mice on high isoflavones had increased *TNF-α* brain gene expression (6 ± 1 and 9 ± 8) compared to their corresponding controls (1.2 ± 0.5, *P* = 0.0079 and 1 ± 0.3, *P* = 0.0079, respectively), high isoflavones decreased *TNF-α* gene expression in the brain of poly(I:C)/swim-treated mice (6 ± 1, *P* = 0.0025) compared to those on low isoflavones (Figure 3A). There was no effect of poly(I:C) treatment on *IL-6* brain gene expression (Figure 3B), but high isoflavones still decreased *IL-6* gene expression in the brain of poly(I:C)/swim-treated mice (0.5 ± 0.1, *P* = 0.0228) compared to those on low isoflavones (2.1 ± 2.4) (Figure 3B).

*KC* gene expression in the brain of poly(I:C)-treated mice on low isoflavones was increased (18 ± 13 and 40 ± 52) compared to their controls (0.9 ± 0.3, *P* = 0.0006 and 1 ± 0.3, *P* = 0.0183, respectively) (Figure 3C). Although poly(I:C)/swim-treated mice on high isoflavones still had increased *KC* gene expression in their brain (2 ± 0.9) compared to control/swim-treated mice (0.6 ± 0.1, *P* = 0.0159), high isoflavones reduced this increase in *KC* gene expression (2 ± 0.9, *P* = 0.0177) compared to poly(I:C)/swim-treated mice on low isoflavones (Figure 3C).

Likewise, *CCL4* gene expression in the brain of poly(I:C)-treated mice on low isoflavones was also increased (6.5 ± 4 and 2.9 ± 1.6) compared to their controls (1 ± 0.3, *P* = 0.0006 and 1 ± 0.4, *P* = 0.0350, respectively) (Figure 3D). Although, poly(I:C)/swim-treated mice on high isoflavones still had increased *CCL4* gene expression (1.8 ± 0.4) compared to control/swim-treated mice (1 ± 0.4, *P* = 0.0317), high isoflavones reduced this increase in *CCL4* gene expression (1.8 ± 0.4, *P* = 0.0101) compared to poly(I:C)/swim-treated mice on low isoflavones (Figure 3D). Poly(I:C) treatment also increased *CCL2, CCL5* and *CXCL10* gene expression in the brains of the mice. High isoflavones reduced this noted *CCL2* brain gene expression increase (Additional file 3).

### Effect of poly(I:C) and isoflavones on skin gene expression of inflammatory mediators

**Figure 4:**
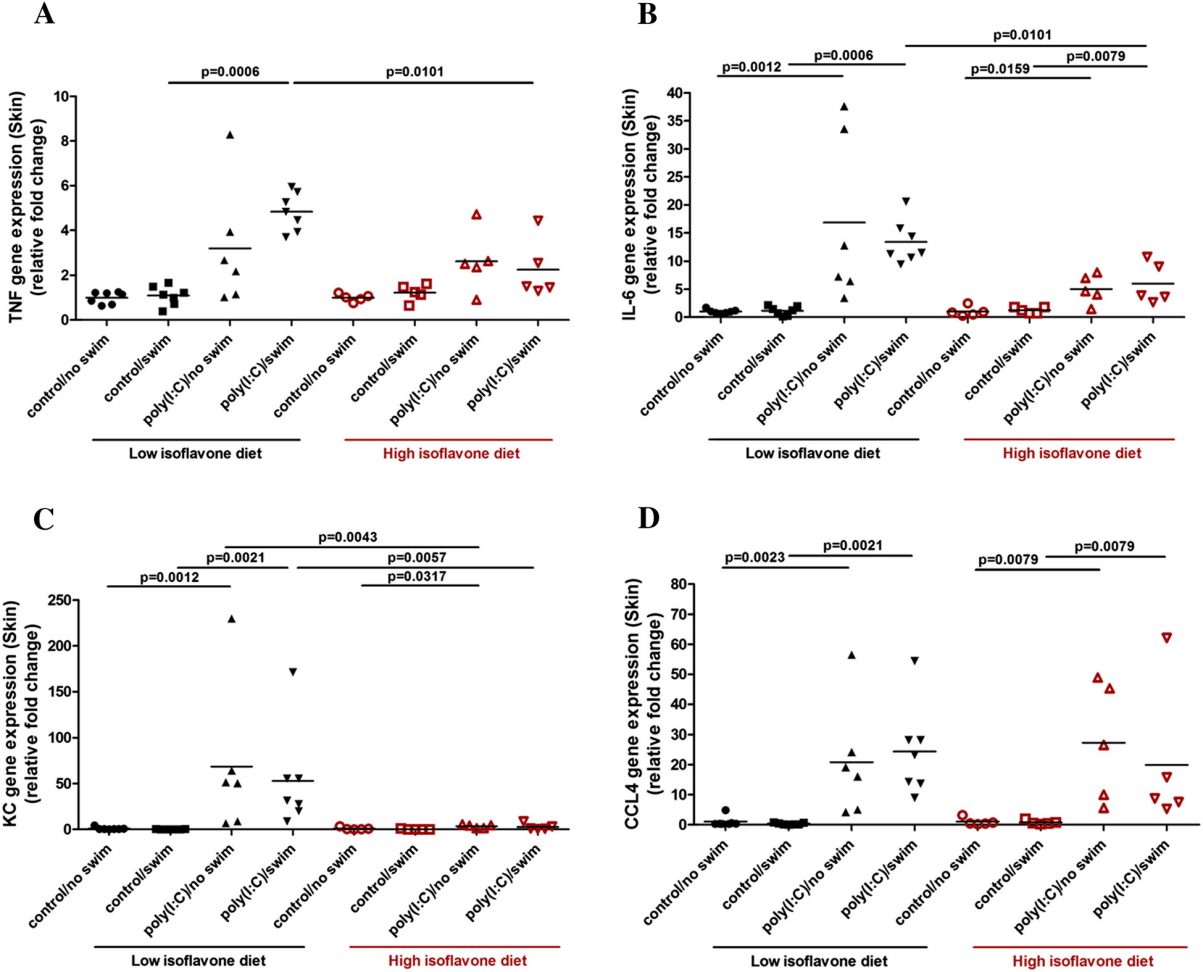
**Polyinosinic:polycytidylic acid (poly(I:C)) and isoflavone effect on skin gene expression of inflammatory mediators.** Mice were provided with either low or high isoflavone diet *ad libitum*. Mice were injected intraperitoneally (ip) with 20 mg/kg poly(I:C) and were forced to swim for 15 minutes. Following this, they were individually placed into specific cages and locomotor activity was monitored overnight. The next day, skin samples were collected and analyzed for *TNF-α* (Figure 4 **A)**, IL-6 (Figure 4 **B)**, *KC* (Figure 4 **C)** and *CCL4* (Figure 4 **D)** skin gene expression using qPCR.

**Figure 5:**
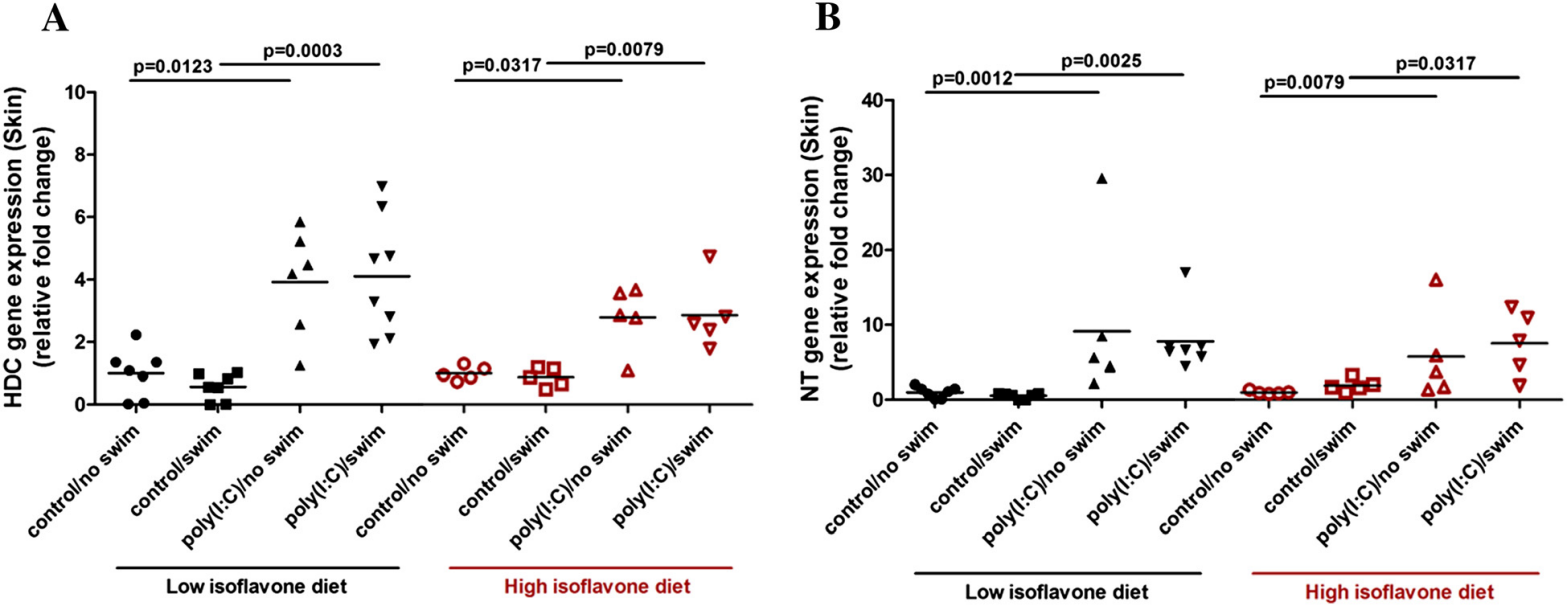
**Polyinosinic:polycytidylic acid** (**poly(I:C)) and isoflavone effect on skin gene expression of inflammatory mediators.** Mice were provided with either low or high isoflavone diet *ad libitum*. Mice were injected intraperitoneally (ip) with 20 mg/kg poly(I:C) and were forced to swim for 15 minutes. Following this, they were individually placed into specific cages and locomotor activity was monitored overnight. The next day, skin samples were collected and analyzed for *HDC* (Figure 5 **A)** and *NT* (Figure 5 **B)** skin gene expression using qPCR.

*TNF-α* (Figure 4A), *IL-6* (Figure 4B), *KC* (Figure 4C), *CCL4* (Figure 4D), as well as *CCL2*, *CCL5* and *CXCL10* (Additional file 5: Table S4) skin gene expression were increased in the poly(I:C)-treated mice. Moreover, *HDC* and *NT* gene expression were also increased in the skin (Figure 5A and B). High isoflavone diet reduced the increased *TNF-α, IL-6* and *KC* skin gene expression.

More specifically, *TNF-α* gene expression in the skin of poly(I:C)/swim-treated mice on low isoflavones, was increased (5 ± 0.9) compared to their controls (1.1 ± 0.4, *P* = 0.0006) (Figure 4A) and high isoflavones decreased this increase in *TNF-α* gene expression (2.3 ± 1.3, *P* = 0.0101) (Figure 4A). Likewise, *IL-6* gene expression in the skin of poly(I:C)-treated mice on low isoflavones was increased (14 ± 4 and 22 ± 16) compared to their controls (0.9 ± 0.8, *P* = 0.0006 and 1 ± 0.4, *P* = 0.0012, respectively) (Figure 4B). Although poly(I:C)-treated mice on high isoflavones still had increased *IL-6* gene expression (5.5 ± 3 and 3.4 ± 1.7) compared to their controls (1.4 ± 0.7, *P* = 0.0079 and 0.7 ± 0.4, *P* = 0.0159, respectively), high isoflavones reduced this increase in the *IL-6* gene expression (5.5 ± 3 *P* = 0.0101) (Figure 4B).

Similarly, *KC* gene expression in the skin of poly(I:C)-treated mice on low isoflavones was also increased (53 ± 55 and 69 ± 83) compared to their controls (0.3 ± 0.2, *P* = 0.0021 and 1 ± 1.5, *P* = 0.0012, respectively) (Figure 4C). High isoflavones reduced this increase in the *KC* gene expression of poly(I:C)-treated mice (2.8 ± 3.5, *P* = 0.0057 and 3.4 ± 1.9, *P* = 0.0043) (Figure 4C).

*CCL4* gene expression was increased in the skin of poly(I:C)-treated mice on low isoflavones (24 ± 15 and 21 ± 19) compared to their controls (0.4 ± 0.3, *P* = 0.0021 and 1 ± 1.7, *P* = 0.0023, respectively) (Figure 4D). *CCL4* gene expression was also increased in the skin of poly(I:C)-treated mice on high isoflavones (20 ± 24 and 27 ± 20) compared to their controls (0.8 ± 0.7, *P* = 0.0079 and 1 ± 1.3, *P* = 0.0079, respectively) and high isoflavones did have an effect on reducing this increase.

In the same way, *HDC* gene expression was increased in the skin of poly(I:C)-treated mice on low isoflavones (4 ± 2 and 4 ± 2) compared to their controls (0.6 ± 0.4, *P* = 0.0003 and 1 ± 0.8, *P* = 0.0123, respectively) (Figure 5A). *HDC* gene expression was also increased in the skin of poly(I:C)-treated mice on high isoflavones (3 ± 1.1 and 3 ± 1) compared to their controls (0.9 ± 0.3, *P* = 0.0079 and 1 ± 0.2, *P* = 0.0317, respectively).

Similar to *HDC* gene expression, *NT* gene expression was increased in the skin of poly(I:C)-treated mice on low isoflavones (8 ± 4 and 9 ± 10) compared to their controls (0.6 ± 0.4, *P* = 0.0025 and 1 ± 0.7, *P* = 0.0012, respectively) (Figure 5B). *NT* gene expression was also increased in the skin of poly(I:C)-treated mice on high isoflavones (8 ± 4, and 6 ± 6) compared to their controls (1.9 ± 0.8, *P* = 0.0317 and 1 ± 0.2, *P* = 0.0079, respectively).

Finally, poly(I:C) treatment also increased *CCL2, CCL5* and *CXCL10* gene expression in the skin of the mice. High isoflavones did not have any statistical effect on *CCL4, CCL2, CCL5, CXCL10, HDC* and *NT* skin gene expression (Additional file 5: Table S4).

## Discussion

Here we show that poly(I:C) significantly reduced locomotor activity over the first 24 hours, in comparison to control mice. Forced swim did not have any effect on its own, but augmented the effect of poly(I:C) on increasing TNF-α, CCL3 and CCL5 serum levels. We also used BALBc mice in an effort to investigate the possibility of strain differences, but there was no difference from our findings with C57BL/6 mice (results not shown).

Use of poly(I:C) increased serum levels of molecules that have been associated with inflammation and fatigue including TNF-α, IL-6, KC, CCL2, CCL3, CCL4, CCL5, and CXCL10. Moreover, poly(I:C) also increased brain and skin gene expression of *TNF-α, IL-6, KC, CCL2, CCL4, CCL5, CXCL10*, while *HDC* and *NT* gene expression were only increased in the skin, suggesting that NT and histamine may explain the skin findings in CFS patients. Forced swim stress had no additional effect to that of poly(I:C), except for augmenting TNF-α serum levels. TNF-α and IL-6 serum levels were actually increased in CFS [20,44]. Such pro-inflammatory mediators can increase blood-brain barrier (BBB) permeability [45] and permit entry of circulating leukocytes leading to brain inflammation. TNF-α was shown to be released along with histamine from rat brain MC [46], and was involved in brain inflammation [47]. MC can also interact with T cells [48-50] and superactivate them through TNF-α [51].

Unlike our present results, one group reported that daily forced swim stress for two to three weeks, with or without an immunological trigger administered on day one (lipopolysaccharide or *Brucella abortus* antigen), induced ‘chronic fatigue’, but only in Albino laca mice [52-55] and Wistar rats [56]. In these papers, behavioral parameters, such as immobility time, time to start grooming after swim stress, roda rod test, and elevated plus maze test were increased indicating post stress fatigue and anxiety. Also, biochemical measurements indicative of brain oxidative stress were increased. In another paper, BALBc mice were injected with *Brucella abortus* and developed decreased running activity that lasted one week [57]. Forced swim of Charles Foster albino rats for twenty-one days also increased immobility time, anxiety as assessed by elevated plus maze test, and brain oxidative stress [58]. These findings maybe due to the differences in the strains and triggers used.

Chemokines interact with their receptors and attract immune cells to inflammation sites [59-61]. CXCR1, CXCR2, CXCR3, CXCR4, CX3CR1, CCR1, CCR3, CCR4 and CCR5 are expressed by human MC of different origins [62]. Stimulated MC release CCL2, CCL3, CCL4 [63]. Murine fetal skin-derived cultured MC (FSMCs) release CCL3, CCL4, CCL5, IL-6 and TNF-α upon TLR3-poly(I:C) activation [64,65]. Peritoneal MC from C57BL/6 mice activated by poly(I:C)-TLR3 have increased CCL5 and CXCL10 expression [66,67].

Here we also show that poly(I:C) with or without NT or CRH did not have any effect on TNF-α, CXCL8 and VEGFα release from human cultured LAD2 MC (results not shown), but increased only *TNF-α* gene expression when used together with CRH, NT, or SP. Poly(I:C) alone did not have any effect on human MC, but increased *TNF-α* gene expression at 24 hours when used together with CRH or NT.

Toll-like receptors’ (TLRs) [68,69] activation is important in the development of innate immunity to invading pathogens, leading to release of different cytokines [64,70,71]. Antigen-mediated MC reactivity is amplified through prolonged TLR-ligand treatment [72].

Human umbilical cord blood-derived MC express TLR-3, activation of which produced IFNα and IFNβ in response to double-stranded RNA [73]. A recent publication reported that MC respond to intracellular, but not extracellular, poly(I:C) by inducing mainly IFNα and TNF-α; moreover, infection of MC with live Sendai virus induces an anti-viral response similar to that of intracellular poly(I:C) [74].

We also used CRHR-1 knockout (KO) mice in order to investigate the possible involvement of CRHR in any stress effect. However, forced swim stress alone did not have an effect and there was no difference using these mice (results not shown). Nevertheless, rats exposed to water immersion stress had a four-fold increase in plasma histamine levels that was absent in W/W^v^ MC-deficient rats [75]. Acute stress also increased serum histamine and IL-6 levels, both of which were also absent in MC deficient W/W^v^ mice [76].

Here we also show that the isoflavones genistein and daidzein reversed the effect of poly(I:C) on mice. Genistein has been reported to attenuate muscle fatigue [77], protect against endothelial barrier dysfunction [78] and suppress LPS-induced inflammatory response in macrophages [79]. Isoflavones also suppress MC expression of the high affinity IgE receptor (FcεRI) [80]. However, isoflavones have estrogenic activity and may not be desirable in certain clinical settings. The flavonoids epigallocatechin, naringin, and curcumin ameliorated ‘chronic fatigue’ [53-56]. Other papers reported similar effects for the *Astragalus* flavonoids [81] and for the olive extract [82].

Flavonoids exert potent anti-inflammatory effects via various pathways [83-87]. A review of human randomized controlled trial studies summarized some significant benefits to cognitive function after isoflavone supplementation [88]: improvements in executive function, working memory and processing speed [88]. Specifically, two studies reported significant effects of 60 mg/day treatment with isoflavones in processing speed and psychomotor speed [89,90].

Quercetin increases exercise tolerance in mice [91]. Oral administration of quercetin leads to accumulation in brain tissue and attenuates the increased oxidative stress in the hippocampus and striatum of rats exposed to chronic forced swimming [92,93]. Quercetin has potent anti-oxidant and anti-inflammatory activity [41,42], and inhibits MC degranulation [94,95], as well as TNF-α, IL-6, and IL-8 secretion [95,96]. Moreover, it reverses acute stress-induced behavioral changes and reduces brain glutathione levels in mice [97].

Recent studies with antigen-stimulated MC show that epigallocatechin gallate (EGCG) inhibits MC degranulation, leukotriene C_4_ secretion, as well as the production of TNF-α, IL-6 and IL-8 [94,96]. The quercetin-related flavone luteolin inhibits MC activation [98] and MC-dependent stimulation of activated T cells [51]. Luteolin also inhibits IL-6 release from microglia cells [99] and from astrocytes [100]. Recent reviews have addressed the possible use of flavonoids for the treatment of CNS diseases [101,102].

Increasing evidence implicates CNS inflammation [103], as well as MC-microglia interactions, in neuropsychiatric diseases [104,105]. MC are important for allergic reactions, but also in immunity [106,107], and inflammatory diseases [15,108]. TLRs have also been implicated in CNS dysfunction through MC and glial activation [104].

## Conclusion

Here we report that treatment of mice with poly(I:C) resulted in reduced locomotor activity and increased inflammatory markers in serum, brain and skin, all of which were reversed by treatment with isoflavones. Our findings also demonstrate significant variability among mouse ‘models’ as was recently reported [109]. There is need for the establishment of a more reliable animal model for CFS. Nevertheless, certain flavonoids appear promising for use in pilot clinical trials with CFS patients.

## Additional files

Additional file 1: Table S1. Additional detailed results for each analyte. Additional file 2: Table S2. Serum levels of inflammatory mediators. Additional file 3:
**Additional results.**
Additional file 4: Table S3. Brain gene expression of inflammatory mediators. Additional file 5: Table S4. Skin gene expression of inflammatory mediators.

## Abbreviations

AD: atopic dermatitis
BBB: blood-brain barrier
CCL: (CC motif) ligand
CDC: US Centers for Disease Control and Prevention
CFS: Chronic Fatigue Syndrome
CNS: central nervous system
CRH: corticotropin-releasing hormone
CRHR-1: CRH receptor-1
CXC: (CXC motif) ligand
EGCG: epigallocatechin gallate
FSMC: murine fetal skin-derived cultured MC
HDC: histidine decarboxylase
HPA: hypothalamic-pituitary-adrenal
PBS/IC: Pelvic Bladder Syndrome/Interstitial Cystitis
IBS: irritable bowel syndrome
IFN: interferon
IL: interleukin
ip: intraperitoneal
KC: keratinocyte-derived chemokine (murine CXCL8/IL-8 homolog)
KO: knockout
LPS: lipopolysaccharide
MC: mast cells
NT: neurotensin
PCR: polymerase chain reaction
Poly(I:C): polyinosinic:polycytidylic acid
SP: substance P
TLRs: Toll-like receptors
TNF: tumor necrosis factor
VEGF: vascular endothelial growth factor
