# Supplementary material for: Isoflavones inhibit poly(I:C)-induced serum, brain, and skin inflammatory mediators - relevance to chronic fatigue syndrome
Source: J Neuroinflammation. 2014 Oct 31;11:168. doi: 10.1186/s12974-014-0168-5 (PMC4236420; doi:10.1186/s12974-014-0168-5)
Supplement: Additional file 1: Table S1. — Additional detailed results for each analyte. [file 12974_2014_168_MOESM1_ESM.doc]

| **Supplemental Table 1** |  |
| --- | --- |
| **Gene of interest/mouse assays** | **Taqman primer/probe set** |
| TNF | Mm00443258_m1 |
| IL-6 | Mm00446190_m1 |
| KC | Mm04207460_m1 |
| CCL2 | Mm00441242_m1 |
| CCL4 | Mm01443111_m1 |
| CCL5 | Mm01302427_m1 |
| CXCL10 | Mm00445235_m1 |
| NT | Mm00481140_ml |
| HDC | Mm00456104_m1 |
| GAPDH | 4352339E-1207040 |
