# Supplementary material for: Isoflavones inhibit poly(I:C)-induced serum, brain, and skin inflammatory mediators - relevance to chronic fatigue syndrome
Source: J Neuroinflammation. 2014 Oct 31;11:168. doi: 10.1186/s12974-014-0168-5 (PMC4236420; doi:10.1186/s12974-014-0168-5)
Supplement: Additional file 2: Table S2. — Serum levels of inflammatory mediators. [file 12974_2014_168_MOESM2_ESM.docx]

**Supplemental Table 2. Serum levels of inflammatory mediators**

| **Conditions** | | **Mediator serum levels (pg/ml)** | | | | | | | |
| --- | --- | --- | --- | --- | --- | --- | --- | --- | --- |
|  |  | **TNFα** | **IL-6** | **KC** | **CCL2** | **CCL3** | **CCL4** | **CCL5** | **CXCL10** |
| **Low isoflavone diet** | **Control/**  **no swim** | 32±0 | 32±0 | 132±52 | 800±0 | 160±0 | 160±0 | 43±14 | 194±30 |
|  | **Control/**  **swim** | 32±0 | 32±0 | 173±43 | 800±0 | 160±0 | 160±0 | 36±7 | 214±42 |
|  | **Poly(I:C)/**  **no swim** | 37±10 | 141±119 | 626±523 | 1470±1023 | 160±0 | 395±245 | 645±438 | 2505±1602 |
|  | **Poly(I:C)/**  **swim** | 55±14 | 229±110 | 735±579 | 3690±2081 | 249±88 | 1256±556 | 1454±554 | 3102±489 |
| **High isoflavone diet** | **Control/**  **no swim** | 32±0 | 32±0 | 140±13 | 800±0 | 160±0 | 160±0 | 34±3 | 138±48 |
|  | **Control/**  **swim** | 32±0 | 32±0 | 100±45 | 800±0 | 160±0 | 160±0 | 33±3 | 152±22 |
|  | **Poly(I:C)/**  **no swim** | 32±0 | 32±0 | 128±43 | 836±81 | 160±0 | 265±111 | 426±298 | 2085±266 |
|  | **Poly(I:C)/**  **swim** | 32±0 | 35±5 | 451±107 | 838±54 | 160±0 | 217±55 | 620±120 | 2362±247 |
