# Supplementary material for: Isoflavones inhibit poly(I:C)-induced serum, brain, and skin inflammatory mediators - relevance to chronic fatigue syndrome
Source: J Neuroinflammation. 2014 Oct 31;11:168. doi: 10.1186/s12974-014-0168-5 (PMC4236420; doi:10.1186/s12974-014-0168-5)
Supplement: Additional file 3: — Additional results. [file 12974_2014_168_MOESM3_ESM.docx]

**Additional File 3**

**ADDITIONAL RESULTS**

**Effect of poly(I:C) and isoflavones on serum inflammatory mediators**

CCL2 serum levels were increased in poly(I:C)-treated mice on low isoflavones (3690±2081 pg/ml and 1470±1023 pg/ml), compared to their controls (800±0 pg/ml, p=0.0002 and 800±0 pg/ml, p=0.0067, respectively). High isoflavones decreased CCL2 serum levels in poly(I:C)/swim-treated mice (838±54 pg/ml, p=0.0054) (Suppl. Table 2).

Similarly, CCL3 serum levels were increased in poly(I:C)/swim-treated mice on low isoflavones (249±88 pg/ml), compared to control/swim-treated mice (160±0 pg/ml, p=0.0056) and high isoflavones reduced this increase in the CCL3 serum levels (160±0 pg/ml, p=0.0307) (Suppl. Table 2).

CCL5 serum levels were also increased in poly(I:C)-treated mice on low isoflavones (1454±554 pg/ml and 645±438 pg/ml), compared to their controls (36±7 pg/ml, p=0.0021 and 43±14 pg/ml, p=0.0098, respetively). Swim further increased CCL5 serum levels (1454±554 pg/ml, p=0.0221). Although poly(I:C)-treated mice on high isoflavones still had increased CCL5 serum levels (620±120 pg/ml and 426±298 pg/ml), compared to their controls (33±3 pg/ml, p=0.0097 and 34±3 pg/ml, p=0.0179, respectively), high isoflavones reduced this increase in the CCL5 serum levels (620±120 pg/ml, p=0.0480) (Suppl. Table 2).

Likewise, CXCL10 serum levels were increased in poly(I:C)-treated mice on low isoflavones (3102±489 pg/ml and 2505±1602 pg/ml), compared to their controls (214±42 pg/ml, p=0.0006 and 194±30, p=0.0246, respectively). Poly(I:C)-treated mice on high isoflavones still had increased levels (2362±247 pg/ml and 2085±266 pg/ml), compared to their corresponding controls (152±22 pg/ml, p=0.0079 and 138±48 pg/ml, p=0.0159, respectively). However, high isoflavones reduced this increase in the CXCL10 serum levels in poly(I:C)/swim-treated mice, but also control/swim and control/no swim-treated mice (2362±247 pg/ml, p=0.0480; 152±22 pg/ml, p=0.0025; and 138±48 pg/ml, p=0.0303, respectively) (Suppl. Table 2).

**Effect of poly(I:C) and isoflavones on brain gene expression of inflammatory mediators**

CCL2 gene expression in the brain of poly(I:C)-treated on low isoflavones was increased (63±40 and 32±39), compared to their controls (1±0.3, p=0.0006 and 1±0.3 p=0.0012, respectively). Although poly(I:C)-treated mice on high isoflavones had increased CCL2 gene expression (13±6 and 18±12), compared to their controls (1.1±0.3, p=0.0079 and 1±0.3, p=0.0079, respectively), high isoflavones reduced this increase in the CCL2 gene expression in the brain of poly(I:C)/swim-treated mice (13±6, p=0.0025) (Suppl. Table 3).

CCL5 gene expression in the brain of poly(I:C)-treated mice on low isoflavones was increased (42±12 and 42±16), compared to their controls (1.1±0.8, p=0.0006 and 1±0.4 p=0.0012, respectively). Poly (I:C)-treated mice on high isoflavones still had increased CCL5 gene expression (47±16 and 46±25), compared to their controls (1.3±0.5, p=0.0079 and 1±0.2, p=0.0079, respectively). There was no effect of high isoflavones on poly(I:C)/-treated mice compared to their respective groups on low isoflavones (Suppl. Table 3).

CXCL10 gene expression in the brain of poly(I:C)-treated mice on high isoflavones was increased (31±8 and 39±16), compared to their controls (1.6±0.7, p=0.0079 and 1±0.2, p=0.0079, respectively) and there was no statistical difference in any conditions tested with the use of high isoflavones (Suppl. Table 3).

**Effect of poly(I:C) and isoflavones on skin gene expression of inflammatory mediators**

CCL2 gene expression was increased in the skin of poly(I:C)-treated mice on low isoflavones (8±6, and 10±7), compared to their controls (0.8±0.4 p=0.0021 and 1±1.3, p=0.0047, respectively) and in the skin of poly(I:C)-treated mice on high isoflavones (7±6 and 8±6), compared to their controls (0.8±0.4, p=0.0079 and 1±0.5, p=0.0079, respectively) (Suppl. Table 4).

CCL5 gene expression was increased in the skin of poly(I:C)-treated mice on low isoflavones (78±29 and 87±46), compared to their controls (0.8±0.5, p=0.0006 and 1±0.4, p=0.0012, respectively) and of poly(I:C)-treated mice on high isoflavones (57±45 and 77±41), compared to their controls (1.1±1, p=0.0079 and 1±0.4, p=0.0079, respectively) (Suppl. Table 4).

CXCL10 gene expression was increased in the skin of poly(I:C)/no swim-treated mice on low isoflavones (19±14), compared to their controls (1±0.2, p=0.0012) and of poly(I:C)-treated mice on high isoflavones (28±17 and 25±17), compared to their controls (1.1±0.5, p=0.0079 and 1±1, p=0.0079, respectively) (Suppl. Table 4).

Overall, high isoflavones did not have any statistical effect on CCL2, CCL5 and CXCL10 skin gene expression (Suppl. Table 4).
