# Supplementary material for: Isoflavones inhibit poly(I:C)-induced serum, brain, and skin inflammatory mediators - relevance to chronic fatigue syndrome
Source: J Neuroinflammation. 2014 Oct 31;11:168. doi: 10.1186/s12974-014-0168-5 (PMC4236420; doi:10.1186/s12974-014-0168-5)
Supplement: Additional file 4: Table S3. — Brain gene expression of inflammatory mediators. [file 12974_2014_168_MOESM4_ESM.docx]

**Supplemental Table 3. Brain gene expression of inflammatory mediators**

| **Conditions** | | **Relative Fold Change** | | | | | | | | |
| --- | --- | --- | --- | --- | --- | --- | --- | --- | --- | --- |
|  |  | **TNFα** | **IL-6** | **KC** | **CCL2** | **CCL4** | **CCL5** | **CXCL10** | **NT** | **HDC** |
| **Low isoflavone diet** | **Control/**  **no swim** | 1±0.7 | 1±0.8 | 1±0.3 | 1±0.3 | 1±0.4 | 1±0.4 | 1±0.5 | 1±1.2 | 1±0.5 |
|  | **Control/**  **swim** | 1.1±0.9 | 1±0.8 | 0.9±0.3 | 1±0.3 | 1±0.3 | 1.1±0.8 | 1.2±0.8 | 0.9±0.6 | 1.1±0.5 |
|  | **Poly(I:C)/**  **no swim** | 12±9 | 1.2±1.3 | 40±52 | 32±39 | 2.9±1.6 | 42±16 | 19±20 | 0.7±0.5 | 0.9±0.5 |
|  | **Poly(I:C)/**  **swim** | 22±7 | 2.1±2.4 | 18±13 | 63±40 | 6.5±4 | 42±12 | 34±26 | 1.2±1.2 | 1±0.6 |
| **High isoflavone diet** | **Control/**  **no swim** | 1±0.3 | 1±0.2 | 1±0.1 | 1±0.3 | 1±0.3 | 1±0.2 | 1±0.2 | 1±0.2 | 1±0.3 |
|  | **Control/**  **swim** | 1.2±0.5 | 0.9±0.3 | 0.6±0.1 | 1.1±0.3 | 1±0.4 | 1.3±0.5 | 1.6±0.7 | 0.9±0.3 | 1.4±0.4 |
|  | **Poly(I:C)/**  **no swim** | 9±8 | 0.5±0.1 | 1.5±0.7 | 18±12 | 2.5±0.9 | 46±25 | 39±16 | 0.8±0.2 | 1.4±0.6 |
|  | **Poly(I:C)/**  **swim** | 6±1 | 0.5±0.1 | 2±0.9 | 13±6 | 1.8±0.4 | 47±16 | 31±8 | 1.3±0.3 | 1±0.2 |
