# Supplementary material for: Isoflavones inhibit poly(I:C)-induced serum, brain, and skin inflammatory mediators - relevance to chronic fatigue syndrome
Source: J Neuroinflammation. 2014 Oct 31;11:168. doi: 10.1186/s12974-014-0168-5 (PMC4236420; doi:10.1186/s12974-014-0168-5)
Supplement: Additional file 5: Table S4. — Skin gene expression of inflammatory mediators. [file 12974_2014_168_MOESM5_ESM.docx]

**Supplemental Table 4. Skin gene expression of inflammatory mediators**

| **Conditions** | | **Relative Fold Change** | | | | | | | | |
| --- | --- | --- | --- | --- | --- | --- | --- | --- | --- | --- |
|  |  | **TNFα** | **IL-6** | **KC** | **CCL2** | **CCL4** | **CCL5** | **CXCL10** | **NT** | **HDC** |
| **Low isoflavone diet** | **Control/**  **no swim** | 1±0.3 | 1±0.4 | 1±1.5 | 1±1.3 | 1±1.7 | 1±0.4 | 1±0.2 | 1±0.7 | 1±0.8 |
|  | **Control/**  **swim** | 1.1±0.4 | 0.9±0.8 | 0.3±0.2 | 0.8±0.4 | 0.4±0.3 | 0.8±0.5 | 1.2±0.6 | 0.6±0.4 | 0.6±0.4 |
|  | **Poly(I:C)/**  **no swim** | 3.2±2.7 | 22±16 | 69±83 | 10±7 | 21±19 | 87±46 | 19±14 | 9±10 | 4±2 |
|  | **Poly(I:C)/**  **swim** | 5±0.9 | 14±4 | 53±55 | 8±6 | 24±15 | 78±29 | 29±22 | 8±4 | 4±2 |
| **High isoflavone diet** | **Control/**  **no swim** | 1±0.2 | 0.7±0.4 | 1±1.4 | 1±0.5 | 1±1.3 | 1±0.4 | 1±1 | 1±0.2 | 1±0.2 |
|  | **Control/**  **swim** | 1.2±0.4 | 1.4±0.7 | 0.3±0.5 | 0.8±0.4 | 0.8±0.7 | 1.1±1.0 | 1.1±0.5 | 1.9±0.8 | 0.9±0.3 |
|  | **Poly(I:C)/**  **no swim** | 2.6±1.4 | 3.4±1.7 | 3.4±1.9 | 8±6 | 27±20 | 77±41 | 25±17 | 6±6 | 3±1 |
|  | **Poly(I:C)/**  **swim** | 2.3±1.3 | 5.5±3 | 2.8±3.5 | 7±6 | 20±24 | 57±45 | 28±17 | 8±4 | 3±1.1 |
